# Graphene-All-Around Cobalt Interconnect with a Back-End-of-Line Compatible Process

**DOI:** 10.1021/acs.nanolett.3c04833

**Published:** 2024-01-31

**Authors:** Chi-Yuan Kuo, Jia-Heng Zhu, Yun-Ping Chiu, I-Chih Ni, Mei-Hsin Chen, Yuh-Renn Wu, Chih-I Wu

**Affiliations:** † Graduate Institute of Photonics and Optoelectronics and Department of Electrical Engineering, National Taiwan University, Taipei 106, Taiwan; ‡ Department of Electro-Optical Engineering, National Taipei University of Technology, Taipei 106, Taiwan

**Keywords:** Co interconnects, graphene-all-around, BEOL compatible, electromigration, reliability

## Abstract

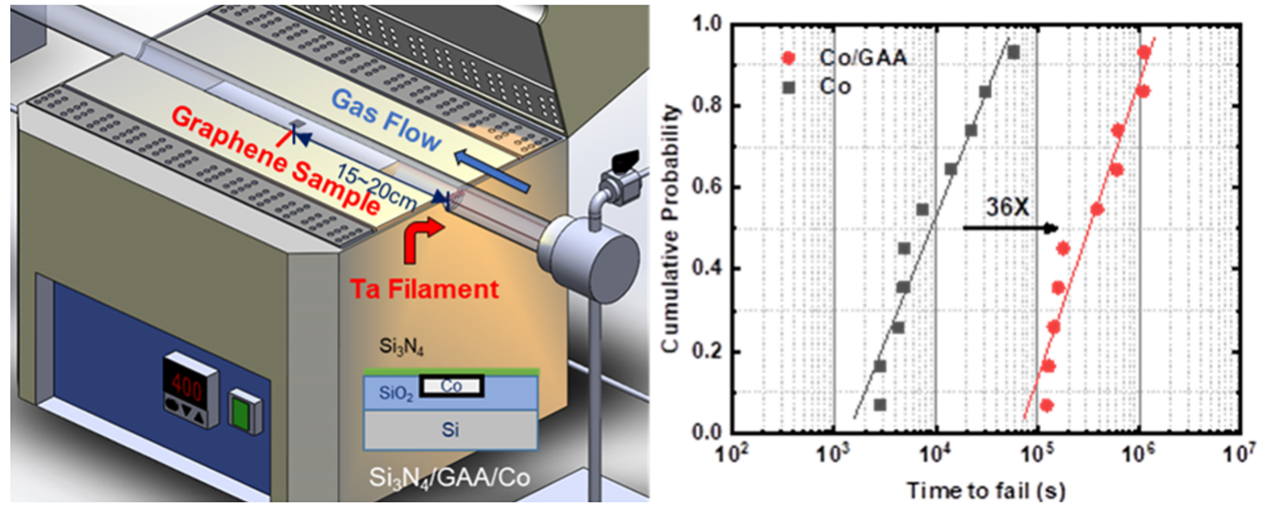

The graphene-all-around (GAA) structure has been verified to grow directly at 380 °C using hot-wire chemical vapor deposition, within the thermal budget of the back end of the line (BEOL). The cobalt (Co) interconnects with the GAA structure have demonstrated a 10.8% increase in current density, a 27% reduction in resistance, and a 36 times longer electromigration lifetime. X-ray photoelectron spectroscopy and density functional theory calculations have revealed the presence of bonding between carbon and Co, which makes the Co atom more stable to resist external forces. The ability of graphene to act as a diffusion barrier in the GAA structure was confirmed through time-dependent dielectric breakdown measurement. The Co interconnect within the GAA structure exhibits enhanced electrical properties and reliability, which indicates compatibility applications as next-generation interconnect materials in CMOS BEOL.

The continuous scaling of semiconductor devices has enabled the integration of increasing numbers of transistors on a single chip, leading to higher performance and functionality.^1^ Copper (Cu) interconnects have been widely used in the semiconductor industry due to their excellent electrical conductivity and low resistivity. However, as the size of the devices continues to shrink, the interconnects that connect the transistors and other components face significant challenges. In advanced technology nodes, Cu interconnects encounter several challenges that limit their performance and reliability.^2^ The Cu line resistivity will increase dramatically when the interconnect dimensions shrink below 30 nm.^3^ Another challenge of Cu interconnects was the phenomenon of electromigration (EM), which could lead to interconnect failure and reliability issues.^4,5^ As the feature size of interconnects shrinks, the current density increases, and the dissipation of heat becomes limited, exacerbating the EM effect.^6^ In addition, the surface roughness and grain boundary scattering at the nanoscale also contribute to an increase in resistivity, which further degrades the performance of Cu interconnects.^7−9^ Parasitic capacitance and inductance also pose challenges in increasing signal delay and power consumption.^10−13^

To overcome these challenges, two approaches could be pursued. First, the resistivity of Cu interconnects could be reduced to mitigate the decrease in the Cu EM lifetime. Recently, graphene has been proposed as a capping layer to simultaneously address the issues of resistance and reliability.^4,14−16^ In previous research, graphene showed excellent impermeability,^17^ thermal,^18^ and electrical properties,^14^ which could alleviate the perturbation of Cu ions^19^ and Joule heating effects and provide an additional current conduction path, thereby increasing the interconnect current density. Moreover, graphene capping nanowires revealed a significant reduction in surface scattering, which improved the conductivity of Cu interconnects.^6^ However, graphene was generally grown by high-temperature (∼1000 °C) chemical vapor deposition (CVD) and needed a transfer process, which is not compatible with CMOS technology, in terms of back-end-of-line (BEOL) thermal budget (<400 °C). Second, alternative materials to replace Cu interconnects, such as cobalt (Co), have been proposed and investigated.^3,20−23^ In the small-dimensional regime, an approach was developed to use an initial selection of alternative metals. A smaller product of the mean free path and bulk resistivity indicates a lower resistivity at small scales.^24,25^ In addition, the melting point could also be used to evaluate the EM performance, indicating that Co had better resistivity and resistance to EM effects than copper at small scales.^26^ As the critical dimension (CD) scaling is <10 nm, the Co line resistivity will cross over with Cu^13^ because the electron mean free path of Co is shorter than Cu, which reduces surface scattering in small trenches. Therefore, Co interconnects are considered to be promising candidates for future interconnects in advanced technology nodes.

In this paper, we integrated the two solutions mentioned above. We designed a process, hot-wire chemical vapor deposition (HW-CVD), which could directly grow graphene that satisfies the BEOL thermal budget. The graphene all around the (GAA) structure was achieved by using Co, which had high carbon solubility catalysts (∼0.13 atom % at 700 °C).^27^ By employing the GAA structure, the resistivity (ρ) of the cobalt interconnect was further reduced by ∼27%, and the breakdown current density (*J*_BR_) was increased by ∼10.8%. Compared with the cobalt wire without encapsulated graphene, the failure time was increased by 36 times. Furthermore, to explore the mechanism behind the Co-wire reliability improvement with graphene, a density functional theory (DFT) calculation was used to verify the bonding between the carbon and Co atom.

Figure 1a shows the HW-CVD setup and the inset of the filament particle working. The HW-CVD process employs a hot filament (tantalum wire, 0.25 mm diameter) placed upstream of the gas flow, with methane (CH_4_) as the carbon source precursor, and carrier gases include hydrogen (H_2_) and argon (Ar). When methane flows through the hot filament, it is thermally pyrolyzed by the high temperature and then reacts with the target substrate in the furnace to grow graphene directly.^28^ The purpose of the furnace was to reduce the level of recombination of hydrogen and carbon atoms. The hot filament was used to enhance the dehydrogenation of the carbon source precursor, and the furnace was used to slow recombination reactions. Because the sample was placed 15–20 cm from the hot filament and not affected by its high temperature, the overall process temperature depends on the temperature set by the furnace. Thus, the process temperature could be effectively controlled and made compatible with BEOL. Furthermore, at this distance, it prevents excessive carbon adsorption on the cobalt, thereby promoting the growth of the quality and more uniform graphene. A schematic diagram of the GAA single damascene structure is shown in the inset of Figure 1b, where high-carbon solubility metal Co was used as the interconnect material. Using HW-CVD, the pyrolyzed carbon rate could increase at a low temperature (380 °C), allowing carbon to reach supersaturation and diffusion into Co.^29^ Upon cooling, carbon precipitated because of the reduction in carbon solubility, finally leading to the GAA structure. The schematic in Figure S1 shows the condition of the synthesized GAA structure determined by HW-CVD at BEOL compatible temperatures. Raman spectroscopy was preliminarily used to examine the quality of the graphene growth by HW-CVD. The legend in Figure 1b denotes the graphene growth on the interconnect top and bottom. The presence of clear D, G, and 2D peaks indicates the existence of graphene, and apparently, the two-dimensional (2D) peak demonstrates crystallized carbon instead of amorphous carbon. Due to the low-temperature growth conditions, numerous small fragments comprising multilayer graphene are formed, leading to weaker 2D peaks compared to those from high-temperature growth.^30^ To verify the feasibility of achieving the GAA structure through carbon diffusion, the surface graphene was removed using hydrogen plasma, followed by wet etching with nitric acid (HNO_3_, 19%, and surfactant) to remove the cobalt. Then, the graphene on SiO_2_ was measured by Raman spectroscopy, which could also clearly reveal a 2D peak, indicating that the diffusion of carbon into Co had grown as graphene at the bottom. Figure S2 benchmarks the temperature of graphene growth on various interconnect metal wires in our work and previous studies.^6,14−16,31−36^ Our report demonstrates the lowest process temperature for graphene growth and the ability to directly synthesize graphene all around a Co wire. These results indicate that HW-CVD could effectively meet the requirements for direct growth and thermal budgets in BEOL. Graphene-all-around Co wire was observed in high-resolution transmission electron microscopy (HRTEM) images, as shown in Figure 1c. After graphene had grown for 15 min, it was evident that graphene successfully encapsulated the Co wire to achieve a GAA structure. The thickness of the graphene was approximately 2.1, 2.1, and 1.7 nm at the top, side, and bottom, respectively, with the number of layers ranging from five to six. Hence, the HW-CVD process was suitable for fabricating the Co–graphene GAA interconnects structure. To gain insight into the GAA achieved by the crystal structure, X-ray diffraction (XRD) analysis was conducted on the cobalt interconnect annealing step. The XRD result revealed a majority HCP (002) grain orientation in the Co interconnect,^37^ which provided proper nucleation sites for graphene^38^ (Figure S3). Additionally, to mitigate oxidation, a H_2_ annealing step was performed prior to the growth of graphene. The absence of characteristic cobalt oxide (CoO) peaks in the XRD spectrum^39^ indicates effective elimination of the oxide layer.

**Figure 1:**
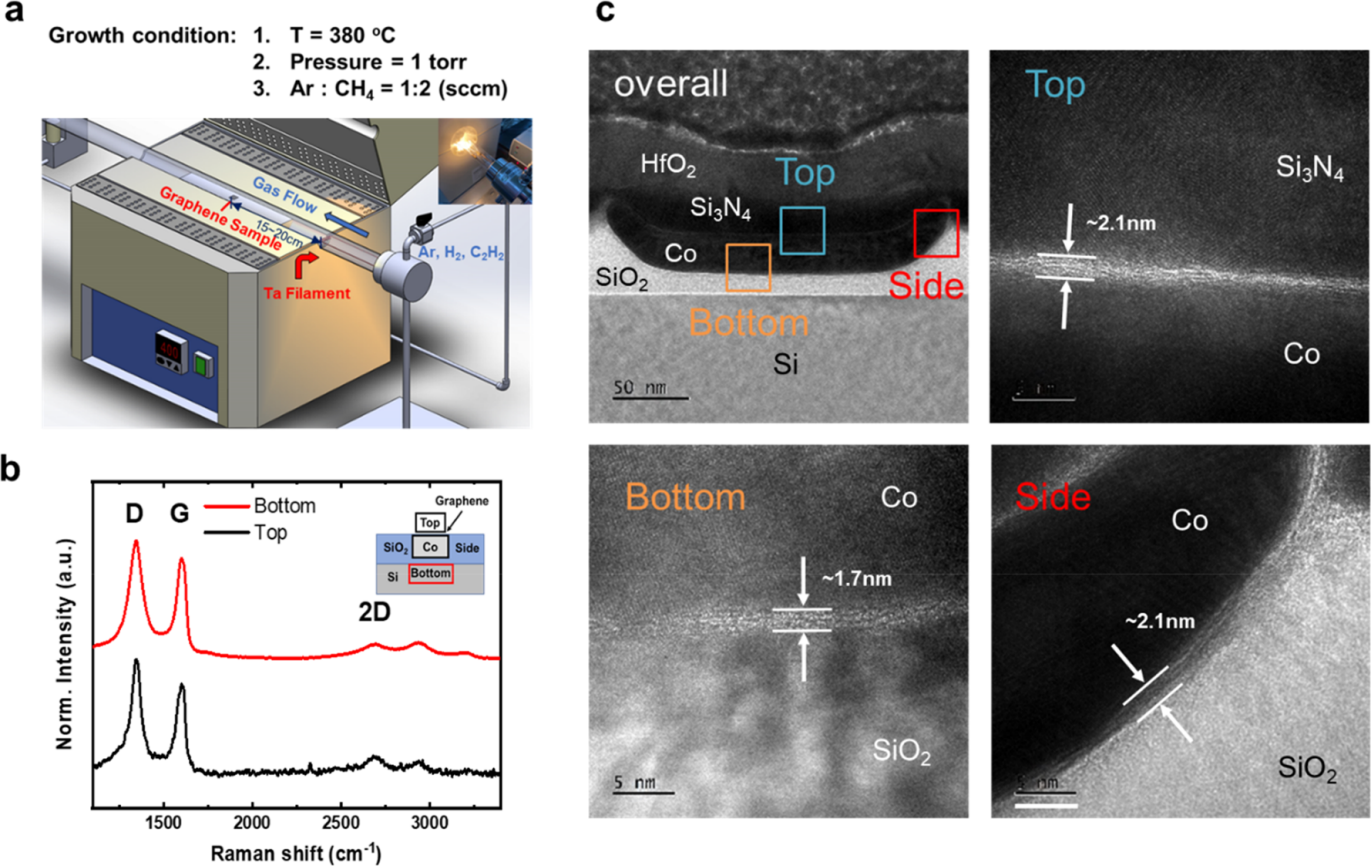
(a) Schematic of the HW-CVD process setup. The inset shows an actual photo of the filament during operation. (b) Raman spectrum of graphene in the GAA structure. The inset shows the schematic of the GAA structure in a single damascene. (c) HRTEM images of graphene all around the structure on the top, bottom, and side in a single damascene Co interconnect.

The electrical properties of the GAA structure (Si_3_N_4_/GAA/Co) were characterized and compared with those of Si_3_N_4_-capped amorphous carbon (Si_3_N_4_/a-c/Co) and annealed Co (Si_3_N_4_/Co) interconnects to demonstrate the advantages of the GAA structure. The differences in growth between annealed Co and the GAA structure come from the absence of a carbon source in annealed Co. The a-c case means the Co wire went through the same process as the GAA structure but without filament aid. A 120 nm thick silicon nitride (Si_3_N_4_) thin film was deposited as an insulating capping layer to isolate moisture, by using PECVD. Figure 2a illustrates the breakdown current density measurement method, where the breakdown current is the maximum current density that the Co wire can withstand under continuous voltage stress. The wire with the GAA structure had the highest *J*_BD_ value of 73.7 MA/cm^2^. Compared to Si_3_N_4_/Co, Si_3_N_4_/a-c/Co exhibits only a slight improvement in current density. In addition, the resistivity was measured using the four-probe method to minimize the influence of contact resistance, which is shown in the inset of Figure 2b. The annealed case means the Co wires went through the same process without a carbon source. The annealed Co interconnects (Si_3_N_4_/Co) showed a resistivity (ρ) of 13.7 μΩ cm and a breakdown current density (*J*_BR_) of 66.5 MA/cm^2^ (Figure 2b,c). The Si_3_N_4_/a-c/Co interconnect showed a ρ of 12.6 μΩ cm and a breakdown current density (*J*_BR_) of 67.7 MA/cm^2^, which is slightly lower than that of the Si_3_N_4_/Co interconnect. In contrast, there is a noticeable performance improvement wherein ρ was reduced to 10 μΩ cm and *J*_BR_ increased to 73.7 MA/cm^2^ for the GAA structure Co interconnects. Simultaneously, it can be observed that under the GAA structure, there is a smaller interquartile range, indicating a more concentrated distribution in the measurement results. This suggests good process uniformity under the GAA structure. The differences in reduced resistivity and increased breakdown current could be attributed to the reduction in surface scattering,^7−9^ Joule heating effects,^40−42^ and the provision of an additional current path.^6,34^ These results demonstrated that graphene encapsulating with Co interconnects using HW-CVD provided performance enhancement and achieved an 27% improvement in ρ and a 10.8% increase in *J*_BR._ Additionally, Figure 2d shows the relation between the *J*_BR_ and ρ that could use a power law (*J*_BR_ = *A*ρ^–*n*^) to describe the relationship throughout the measurement, where *A* is a fitting parameter and *n* is a power factor.^15,16^ A high *n* value indicated a faster breakdown in the interconnect. The power law fitting yielded *n* values of 0.62, 0.54, and 0.48 for Si_3_N_4_/Co, Si_3_N_4_/a-c/Co, and Si_3_N_4_/GAA/Co, respectively. The resistivity, breakdown current density, and power factor of Si_3_N_4_/GAA/Co interconnects are compared with those of Si_3_N_4_/Co interconnects, indicating that the GAA structure could further enhance the electrical properties of Co interconnects. The performance improvement between Si_3_N_4_/Co and Si_3_N_4_/a-c/Co is not substantial, which could be ascribed to amorphous carbon slightly improving heat dissipation and surface scattering.^43^ Graphene, by contrast, had more excellent electrical and thermal conductivity, which could not only provide the current conduction path but also suppress surface scattering and ensure better heat dissipation. To investigate the breakdown process, Figure S4 shows SEM images of the regions where the breakdown occurred for the Co, a-c/Co, and GAA/Co structures in vacuum, respectively. It could be observed that the breakdown area was largest in GAA/Co, followed by a-c/Co, and finally Co. This suggests that the difference in thermal conductivity leads to a localized breakdown in the annealed Co due to Joule heating effects. However, due to graphene’s excellent thermal conductivity, the breakdown area was dispersed. Additionally, Raman measurements were performed on the breakdown regions, revealing the presence of a significant 2D peak in the graphene even after breakdown, confirming its continued existence after breakdown (Figure S5). Furthermore, due to previous studies that diverge on whether Co interconnects require a diffusion barrier^44,45^ or not,^46^ the TDDB measurements were conducted to evaluate the graphene diffusion barrier property, serving as a barrier layer in GAA structures, which is a standard test commonly employed to assess the reliability of the gate dielectric in semiconductor devices.^47^ The TDDB measurement applied a constant electric field at room temperature across the capacitor structure when graphene was used as a barrier layer; it could reduce the rate of diffusion of the Co ion into the dielectric, thus further enhancing the device lifetime (Figure 3a). The electric field drives Co ions into dielectric, causing accumulation and the formation of the trap-assisted conduction path that leads to device breakdown. Therefore, the absence of a barrier layer makes Co ions easier to diffuse into the dielectric, resulting in a shorter lifetime. Figure 3b shows the TDDB measurement results for various fields with graphene as a cobalt barrier compared to the results without graphene. The time to fail with a probability of 50% (TTF_50%_) was used to evaluate the device lifetime.^47^ The TTF_50%_ of graphene barriers was enhanced from 109 to 642 s (5.9 times) at 6 MV/cm, from 41 to 173 s (4.2 times) at 7MV/cm, and from 13 to 50 s (3.8 times) at 8 MV/cm, showing a significant improvement in the device lifetime. Furthermore, we also achieved direct growth graphene on the dielectric by using HW-CVD in conjunction with ammonia (NH_3_), as shown in Figure S6.

**Figure 2:**
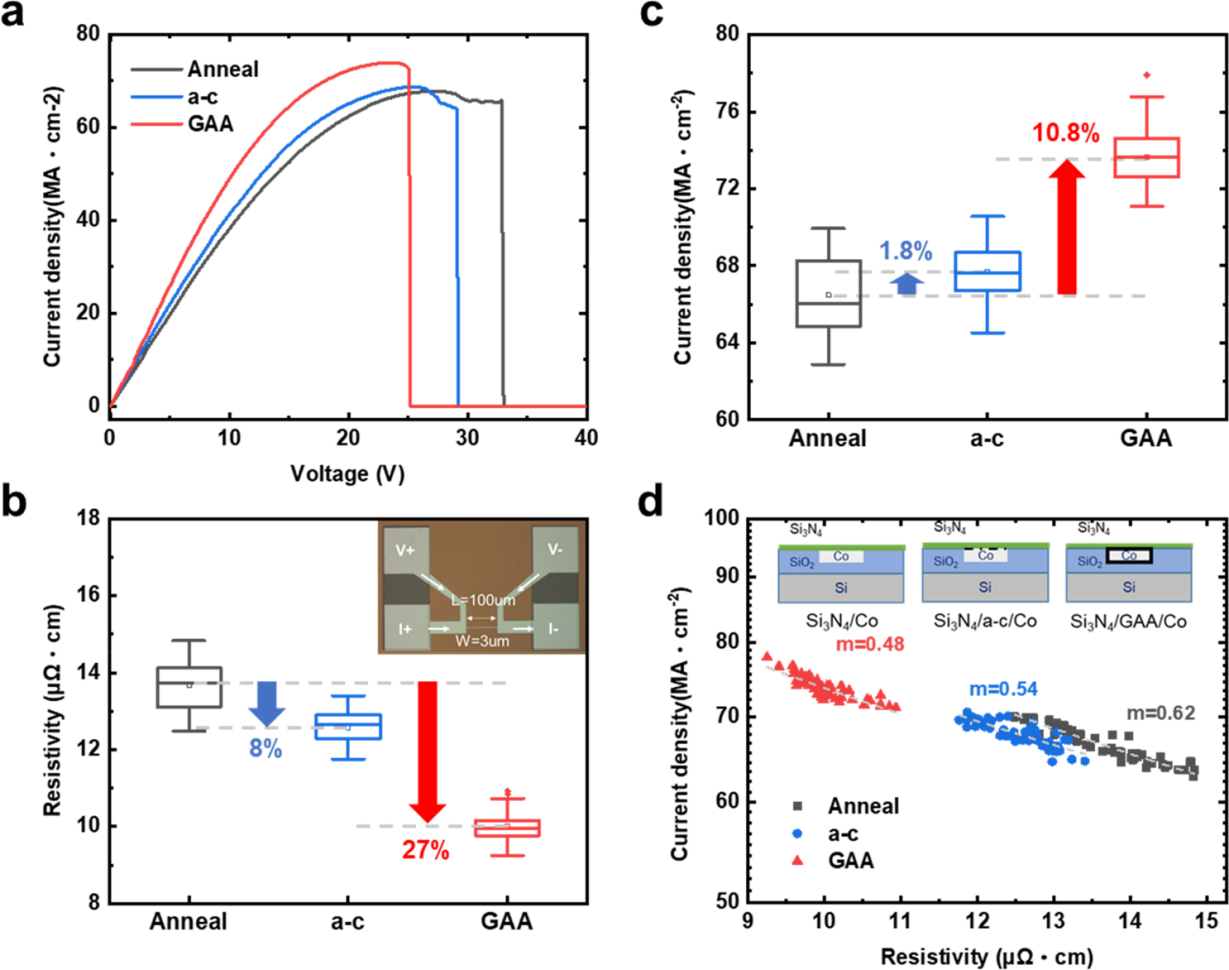
Single damascene interconnect electrical property of the GAA structure. (a) Co-interconnect breakdown current density (*J*_BR_) of annealed (black), capping with a-c (blue), and GAA structure (red). (b) Resistivity of the annealed capping with a-c and GAA structure. The GAA structure shows a 27% reduction in resistivity but capping with a-c of only 8%. (c) Breakdown current density of the annealed capping with a-c and GAA structure. An approximately 10.8% current density increase is observed for the GAA structure, suggesting that graphene provides an additional conduction path. (d) Plots of *J*_BR_ vs ρ of annealed, a-c, and GAA structure interconnects.

**Figure 3:**
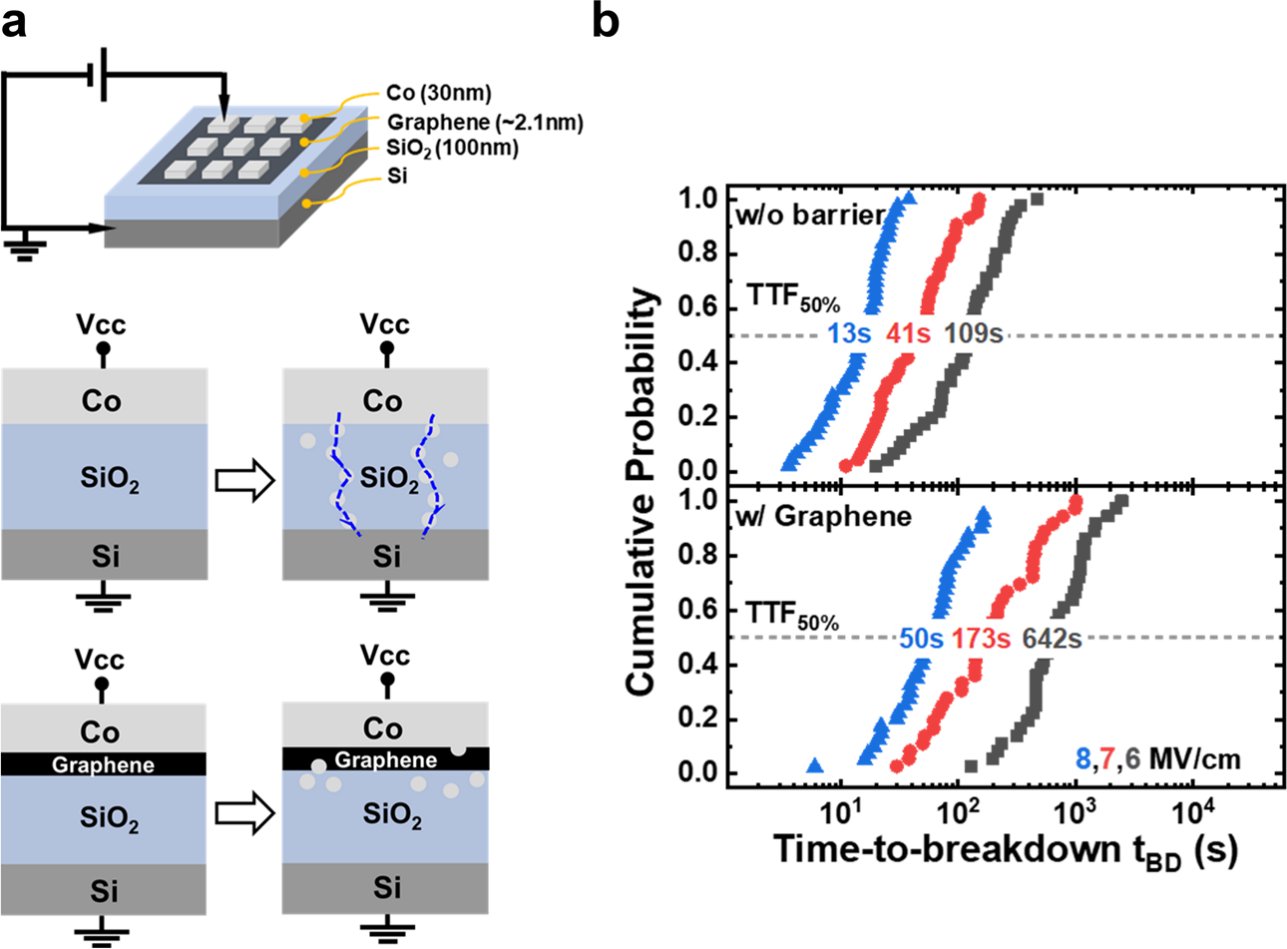
(a) Schematic of TDDB measurements with capacitor structures used to evaluate the GAA structure diffusion barrier property. The bottom schematic shows the diffusion of Co ions into the dielectric of the device with and without graphene. (b) Cumulative distribution of time to breakdown (*t*_BD_) under electric field stresses of 8, 7, and 6 MV/cm. TDDB results without a barrier and device with directly grown graphene in the GAA structure as a diffusion barrier on SiO_2_.

The benchmark for the breakdown current density that could be sustained under various cross-sectional areas is shown in Figure S7.^6,14−16,31,34,36^ One could observe that we achieved a relatively high current density with the GAA structure compared to that with Cu interconnects. This demonstrates that the GAA structure indeed enhanced the performance of Co interconnects, which was even better than that of Cu interconnects. However, the breakdown process for Co interconnects was caused by Joule heating and EM. To understand the role of the EM process, the EM lifetimes of Si_3_N_4_/Co and Si_3_N_4_/GAA/Co were compared under dc current stress, which was performed with an ambient temperature of 200 °C and a current density of 30 MA/cm^2^ in the vacuum. An EM measurement in the vacuum was applied to explore the intrinsic lifetime improvement with the GAA structure interconnect. According to the International Electrotechnical Commission (IEC) constant current EM test criteria, when resistance increases by 10–30%, the corresponding time is defined as the lifetime, and metal wire is considered as open failure. In this research, the time corresponding to a 10% increase in resistance was defined as the lifetime of the Co interconnect failure. Figure 4a shows the mean time to failure (MTTF) of annealed Co and GAA structure Co. The failure time for the Si_3_N_4_/Co interconnect was 8.9 × 10^3^ s because EM in the Co interconnect created voids, which increased the resistance of the interconnects. In contrast, the GAA structure Co interconnect had an increased failure time of ∼3.2 × 10^5^ s, which shows a MTTF 36 times longer than that of annealed Co. To uncover the EM breakdown process with the GAA structure Co interconnect, the resistance change over time was studied, as shown in Figure 4b. It could be observed that both the GAA structure and annealing Co interconnect resistance exhibited an initial increase followed by a decrease, ultimately leading to failure or reaching 10% Δ*R*/*R*_0_. This trend was similar to those of previous studies on single damascene Co interconnects.^3,46^ However, in the case of the GAA structure, a slower increase in the resistance was observed. This result suggests that graphene mitigates the rearrangement of the Co-wire orientations. When orientations with lower strain energy replace them, there is further grain growth along with associated reduction of grain boundary scattering, leading to a decrease in line resistance.^46^ Additionally, the decrease in resistance in the GAA structure was more pronounced compared to that of the annealing Co interconnect. This difference could be attributed to the Co interconnect in the GAA structure, which could reduce not only grain boundary scattering but also surface scattering. The improvement in the lifetime of Co EM may be attributed to the bonding between carbon and cobalt, which captured Co atoms and reduced their level of migration, thereby further enhancing the Co EM lifetime. XPS depth profile analysis with 500 eV Ar ion beam sputtering for 6 min was conducted to verify the interfacial structure of graphene/Co. Before the sputter etching, the peaks of the pristine C 1s profile could be deconvoluted into two peaks that corresponded to sp^2^ and sp^3^ bonding characters at 284.4 and 285.1 eV, respectively.^48^ However, C–Co bonding was detected at ∼283 eV after etching for 6 min as shown in Figure S8a.^49^ In addition, Raman measurements were performed on the location subjected to ion beam etching, as shown in Figure S9. Another observation that supports the persistence of C–C sp^2^ bonding after etching for 6 min was that a significant 2D peak in the Raman spectrum persisted even after ion beam sputtering. This result indicates that ion beam sputtering is effective in reducing the number of graphene layers, leaving at least one layer of graphene for observation of the characteristic Co–C peaks. Moreover, Co 2p_3/2_ and 2p_1/2_ were deconvoluted into three doublets, which appeared at 778.2 eV for the Co 2p_3/2_ peak and 793.1 eV for the Co 2p_1/2_ peak. This spin–orbital splitting between the Co 2p_3/2_ and 2p_1/2_ peaks is 14.9, which could be ascribed to the Co^0+^ metal. The 2p_3/2_ and 2p_1/2_ peaks located at 780.4 and 795.4 eV, respectively, could be observed, which indicated the existence of Co^2+^ ions, as shown in Figure S8b.^50^ The aforementioned XPS results demonstrated that a GAA structure grown through HW-CVD forms bonds between Co and graphene at the interface. This observation could also be reflected in the improvement of the Co EM lifetime. To further understand how the GAA structure mitigate the electromigration, DFT calculations were performed to compare the graphene–Cu (Figure 5a) and graphene–Co (Figure 5b) interfaces.^51−57^ The relaxed interlayer distances are 3.13 Å for the graphene–Cu interface and 2.14 Å for the graphene–Co interface. The graphene–Cu interface exhibits physisorption, evidenced by the preserved Dirac cone near the Fermi level in Figure 5c, indicating van der Waals bonding.^58,59^ In contrast, the graphene–Co interface forms metal carbide bonds and disrupts the graphene electrical structure. This hybridization can be observed in Figure 5d, where the inherent graphene band structure is hardly retained, showcasing chemisorption characteristics. DFT-calculated electrostatic potential plots confirm higher tunneling barriers in the graphene–Cu interface (Figure 5e) due to the lack of electron interaction, while the graphene–Co (Figure 5f) interfaces are barrier-free, indicating chemical bond formation. This bonding, also observed in XPS data, acts as an anchor, aiding Co atoms in resisting electromigration. Consequently, the graphene–Co interface demonstrates better electromigration resistance and a tunneling barrier-free interface, suggesting GAA structure as a superior interconnect material in <10 nm domains.

**Figure 4:**
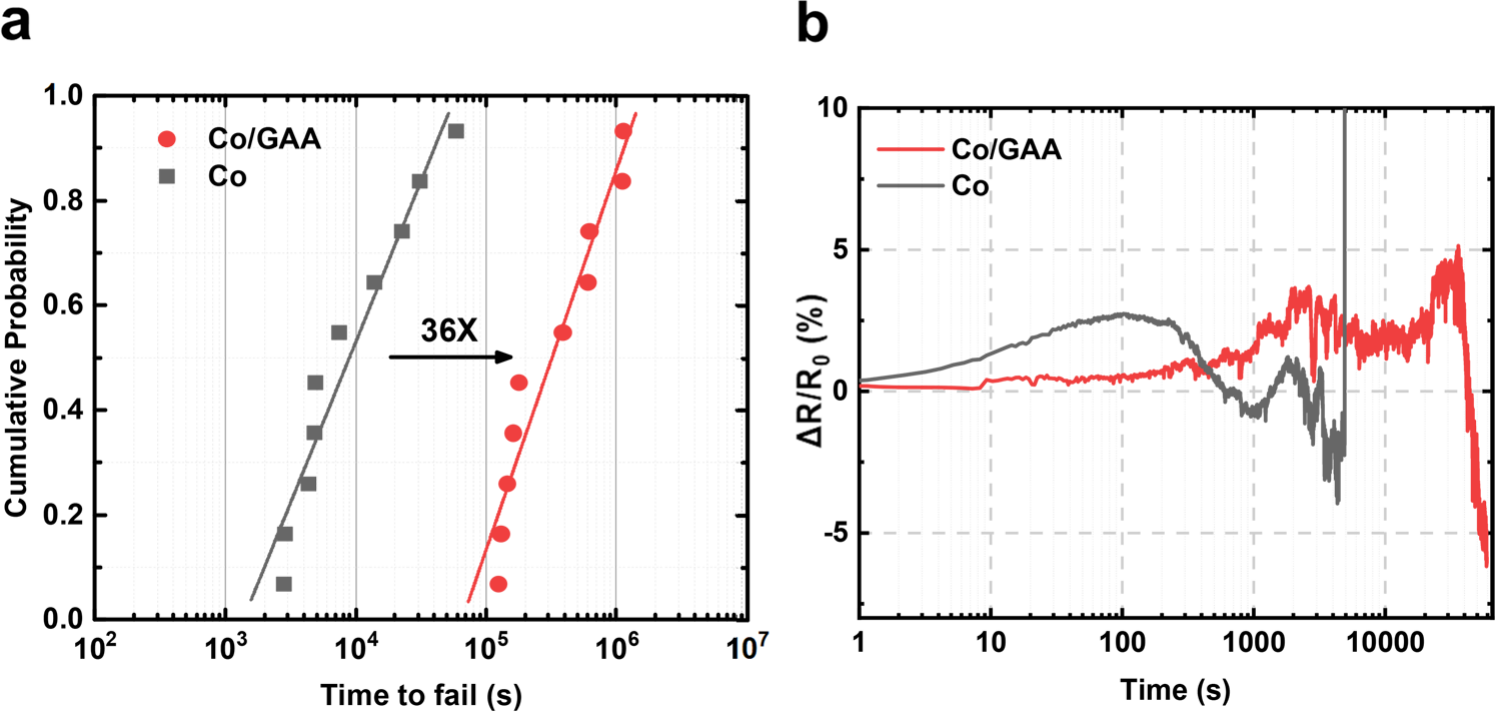
(a) Statistical distribution of the Co EM lifetime for Si_3_N_4_/Co (black) and Si_3_N_4_/GAA/Co (red). The structure is stressed at 200 °C and 30 MA/cm^2^. Co interconnects in the GAA structure show a 36 times longer median time to fail (MTTF). (b) Resistance changes over time during the EM test of annealed Co and with the GAA structure.

**Figure 5:**
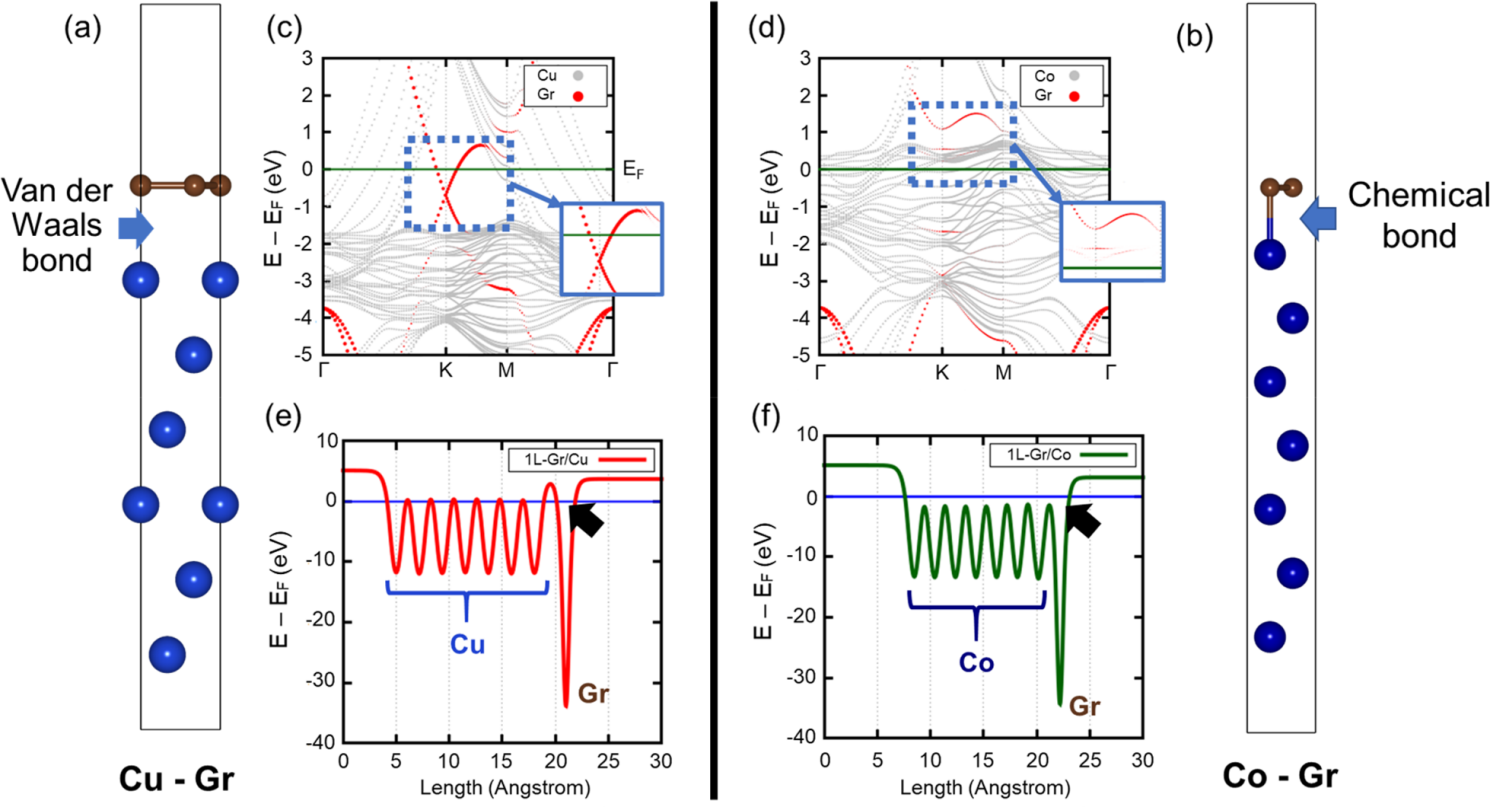
Schematic of the DFT-simulated structures of (a) the Cu–graphene and (b) Co–graphene systems. Projected band structures of (c) Cu–graphene and (d) Co–graphene systems with gray dots for metal and red dots for graphene. The blue dotted box indicates an important hybridization region, and the blue solid box is the projected band structure in which gray dots are removed for more apparent Gr hybridization observations. (e) Cu–graphene and (f) Co–graphene interfaces give the electrostatic potential plots of both systems.

The GAA structure could be directly grown at 380 °C using HW-CVD in conjunction with carbon-dissolved metal cobalt. The Co interconnect in the GAA structure improved the resistivity, breakdown current density, and EM lifetime. These results showed that graphene capping Co interconnects lead to lower electrical resistivity and higher current density in the interconnect due to alleviating Co surface scattering, stronger heat dissipation of graphene, and providing an additional current conduction path. Furthermore, through simulations and XPS measurements, it has been verified that the Co interconnection with the GAA structure exhibited bonding between carbon and cobalt. This bonding helps mitigate cobalt atom migration at the interface, thereby enhancing the EM reliability of the Co interconnection with the GAA structure. This research indicates further feasibility of the application of Co interconnects as a replacement for Cu interconnects when the interconnect shrinks to a small scale

## Supporting Information Available

Description of the material, details of the DFT calculation, and experimental details as well as growth process, benchmark, XRD, SEM, Raman, and XPS data (PDF)
