# Supplementary material for: Graphene-All-Around Cobalt Interconnect with a Back-End-of-Line Compatible Process
Source: Nano Lett. 2024 Jan 31;24(6):2102–9. doi: 10.1021/acs.nanolett.3c04833 (PMC10870778; doi:10.1021/acs.nanolett.3c04833)
Supplement: Supplementary file 1 — nl3c04833_si_001.pdf [file nl3c04833_si_001.pdf]

SUPPORTING INFORMATION

Graphene-All-Around Cobalt Interconnect with Back-End-of-Line Compatible Process

Chi-Yuan Kuo[a], Jia-Heng Zhu[a], Yun-Ping Chiu[a], I-Chih Ni[a], Mei-Hsin Chen[b], Yuh-Renn Wu[a] and Chih-I Wu[a]*

[a]Graduate Institute of Photonics and Optoelectronics and Department of Electrical Engineering, National Taiwan University, Taipei 106, Taiwan

[b]Department of Electro-Optical Engineering, National Taipei University of Technology, Taipei 106, Taiwan

*Email: chihiwu@ntu.edu.tw

S1: DFT calculation details:

The simulation utilizes Quantum ESPRESSO [51] with the projected augmented wave (PAW) pseudopotentials [52] with Generalized Gradient Approximation of Perdew–Burke–Ernzerhof (GGA-PBE) for exchange-correlation approximation [53]. An energy cutoff of 400 eV and 8x8x1 Monkhorst-Pack k point mesh [54] was chosen. The graphene lattice was held at 2.46 Å while the metal lattice was adjusted according to Gr to construct a superlattice. Lattice mismatches for Graphene-Cu and Graphene-Co systems were 3.6% and 2.0%, respectively, where all metals were set to 7 layers [55] to ensure metal bulk characteristics (See **Figure 5**a, 5b). At least 20 Å vacuum regions in the z-direction were included to eliminate the effect of adjacent cells. All simulation models were created for each metal's energetically advantageous surface ((111) surface for Cu and (0001) for Co) with 1x1 metal and 1x1 graphene superlattice construction [56]. The five outward metal layers were fixed during the structural relaxation process. The DFT-D3 correction was used here, as it provides a reasonable van der Waals description and can be used in many scenarios in DFT calculations with good numerical stability [57]. The electronic and atomic structure of graphene-Cu and graphene-Co interfaces were investigated and compared after DFT calculation. The relaxed graphene-Cu and graphene-Co interlayer distances were 3.13 Å and 2.14 Å, respectively. Although GGA-PBE calculations usually overestimate the bonding distance [58], the results can still provide a valid comparative relationship because both systems use the same set of parameters during simulation. Interfaces can be classified into two categories of physisorption and chemisorption interactions by evaluating the orbital hybridization [58, 59]. In our calculation, the graphene-Cu contact can be regarded as a physisorption interface due to preserving the Dirac cone observed near the Fermi level in the projected band diagram in **Figure 5**c. The dot-framed area indicates the existence of the graphene, where the graphene-only band diagram plot (i.e., red dot only) in the same area is shown in the bottom-right blue solid line box. This non-hybridization specifies no chemical bond formation between graphene and Cu. Indicating the absorption phenomenon of graphene

onto Cu is via Van der Waals bonding. Unlike the graphene-Cu system, the graphene-Co interface gives chemisorption-like behavior, which produces metal carbide bonds and disrupts the electrical structure of graphene by strongly hybridizing the orbitals of the metal and graphene. This hybridization can be observed in **Figure 5**d in the solid line box figure, where the inherent graphene band structure is hardly retained, showcasing chemisorption characteristics. This result may also correspond to the observation of the Co-C peak in the XPS data. This additional bonding between the Co and Graphene layer acts like an anchor that benefits the closest Co atoms from resisting the EM effect. In opposition to the graphene-Co system, this phenomenon is impure in the graphene-Cu system because graphene primarily adheres to Cu through physical adsorption. Comparing graphene-Cu and graphene-Co systems, the Graphene-Cu system may endure more EM atom distortion than the graphene-Co system due to the lack of chemical bonding between interfaces, causing faster failure time during operation. The absorption results also correlate well with the interlayer distances since the shorter the interlayer distances, the more likely chemisorption will happen (graphene-Co interlayer distances are shorter than graphene-Cu in this case). The physisorption and chemisorption characteristics can also be verified by the DFT-calculated electrostatic potential plots shown in **Figure 5**e and, 5f for the graphene-Cu and graphene-Co systems, respectively. As the black arrow indicates in **Figure 5**e, the tunneling barrier at the graphene-Cu interface is much higher than the Fermi level. Since there is no chemical connection between the graphene and Cu, the van der Waals gap causes a high tunneling barrier value [60] whereas the formation of the chemical bond in the graphene-Co interface essentially creates a tunnel barrier-free interface, illustrated by the black arrow in **Figure 5**f. Thus, the tunneling barrier in the graphene-Cu interface will mostly confine electrons in the Cu region where the electron may travel into graphene to benefit transport in the graphene-Co interface due to the barrier-free chemisorption interface. All in all, chemical bonding in the graphene-Co system can bring better EM resistance and a tunneling barrier-free interface for benefitting electron transport.

Therefore C-Co bonding could offer an extra binding to make a Co atom more stable and resilient to an external force. Hence, the GAA structure mitigated the EM, resulting in more stable and fastened Co atoms against electrical stress, suggesting that Co may be the better interconnect material in the sub-10 nm domain.

Ref: (60) Zhao, N.; Schwingenschlögl, U. Dipole-induced Ohmic contacts between monolayer Janus MoSSe and bulk metals. *npj 2D Materials and Applications* **2021**, *5* (1), 72.

S2: Experimental details:

Cobalt interconnects were fabricated on the top of an SiO_2 film which was thermally grown to a thickness of 50 nm on a p-type doped silicon (100) wafer. The substrates were cleaned using acetone (C_3H_6O), methanol (CH_3OH), and isopropanol (IPA) with ultrasonic cleaner wash respectively for 10 min. The photoresist (MICROPOSIT S1813G) was spin-coated on SiO_2. Subsequently, the interconnect pattern used mask aligner (Karl Suss MA6 Mask Aligner) lithography with a wavelength of 405 nm. The resultant pattern was then developed using MICROPOSIT MF319. Finally, the single damascene structure was etched by reactive-ion etching (RIE). The etching process utilized a gas mixture of 40 sccm fluoroform (CHF3) and 10 sccm oxygen (O_2) at a pressure of 6.7 Pa and a plasma power of 250 W. With these parameters, the etching rate of SiO_2 film was 8.4 Å/s. Following the etching process, the Co film (30nm) was deposited by electron beam (E-beam) evaporation at a pressure of $5x10^{-6}$ torr. The HW-CVD system is pumped down to a pressure of $1x10^{-3}$ torr using a mechanical pump, followed by purging with 200 sccm Ar, 200 sccm H_2 and 200 sccm CH_4 to ensure quartz tube cleanliness. In addition, all gas flow rates were controlled using computer-controlled Mass Flow Controllers (MFC). In order to characterize the graphene quality, the sample was investigated by Raman spectroscopy (Kymera 328i, Andor) with an excitation wavelength of 532 nm and a laser power of 64 mW and by X-ray photoelectron spectroscopy (XPS) (ULVAC PHI 5000 Versa Probe). The spot size for XPS measurements is 100 µm in diameter for PHI

5000. In the failure analysis process, the areas of interconnect breakdown were captured through scanning electron microscope (SEM) (JSM-7001F, JEOL). The cross-sectional TEM samples were fabricated by focused ion beam (FIB) (FEI Helios) and were investigated by TEM (FEI Talos F200X). To fabricate the metal-oxide-semiconductor (MOS) capacitor structures, a 30nm Co film was deposited by E-beam evaporation. For the TDDB measurements, the graphene was prepared under the same conditions as the interconnect then applied to a constant electric field across the capacitor structure using Keysight B2912A.

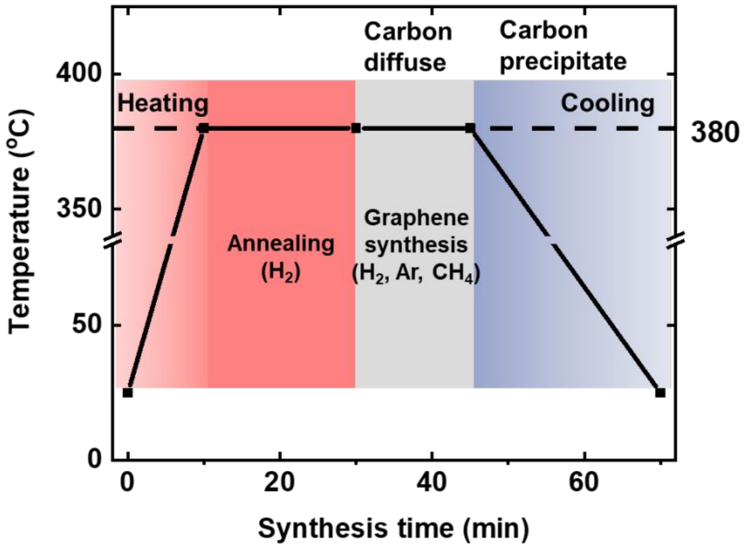

Figure S1. Schematic and condition of the growth process of graphene-all-around Co interconnects using HW-CVD at BEOL-compatible temperature

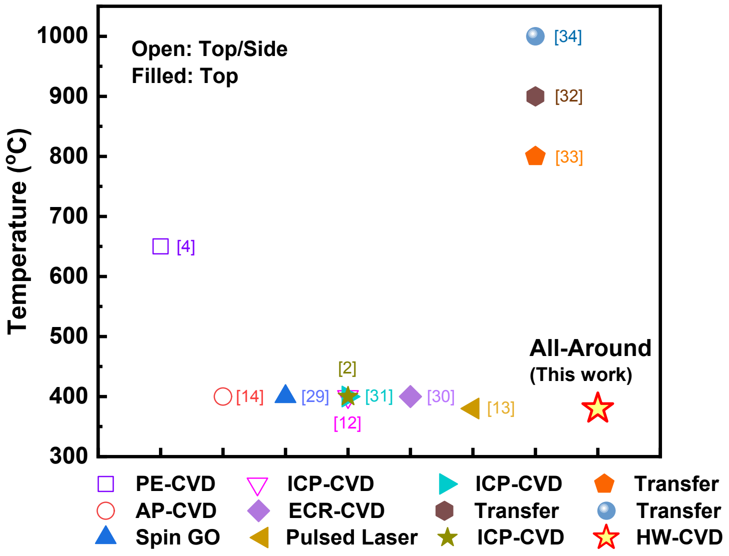

Figure S2. Benchmark of graphene growth temperature on the interconnect. The open and filled symbols are Top/Side and Top of the graphene growth region on the interconnect, respectively.

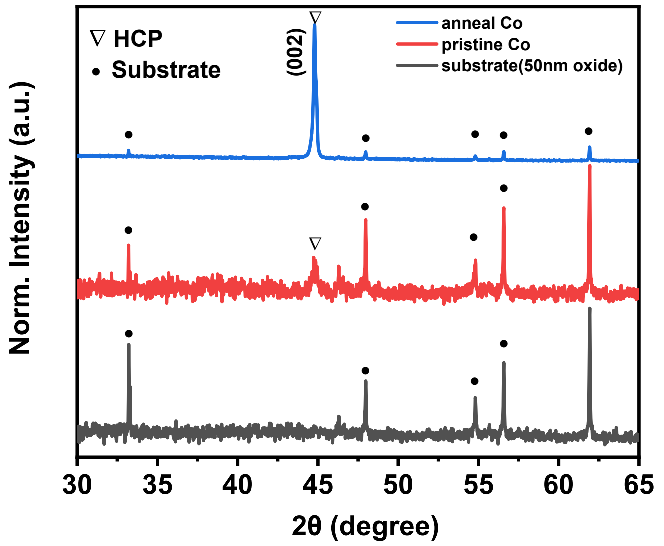

Figure S3. XRD spectrum of Co interconnect

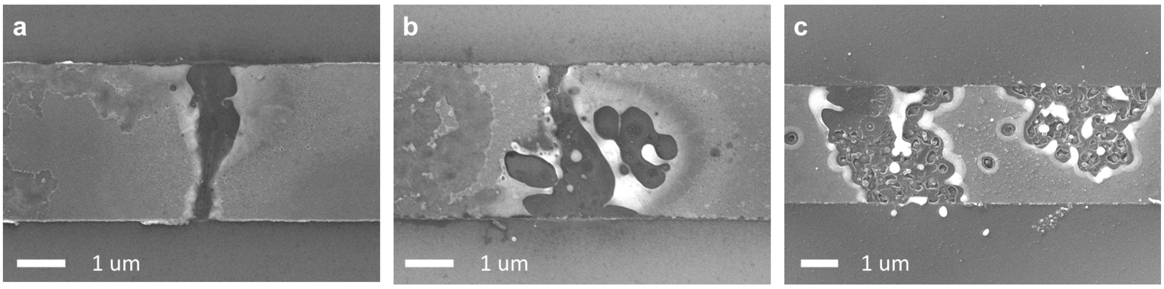

Figure S4. SEM images of breakdown points on (a) annealed Co, (b) A-C/Co, (c)GAA/Co. The GAA/Co structure exhibits smaller scale bars due to its larger breakdown region

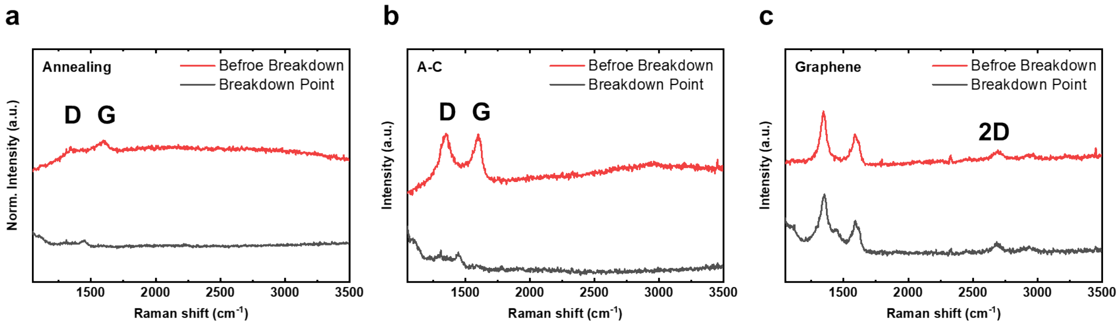

Figure S5. Raman Spectrum of before and after the breakdown points on (a) annealed Co, (b) A-C/Co, (c)GAA/Co.

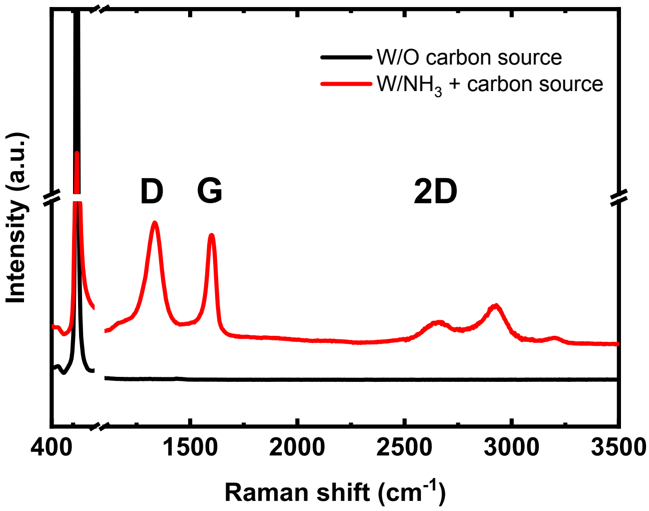

Figure S6. Raman spectrum of graphene direct growth on SiO_2 dielectric by using HW-CVD aid with NH_3.

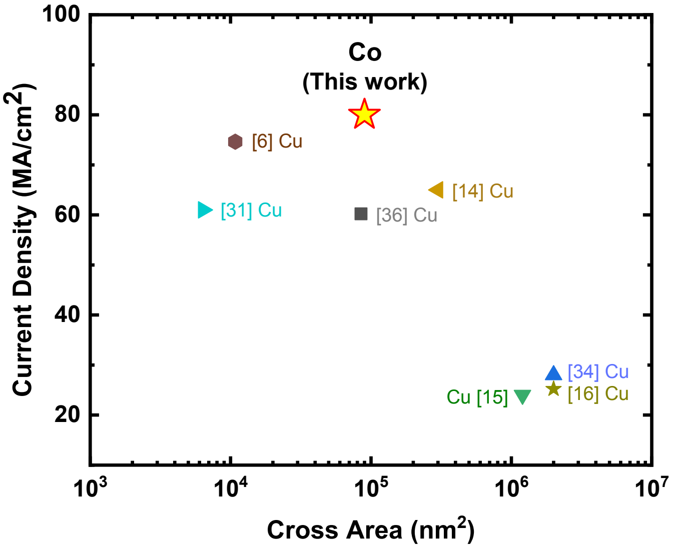

Figure S7. Benchmark of current density in graphene capping interconnects with different cross areas. Among the different research considered in this study, all metal interconnects except for this work refers to copper interconnect

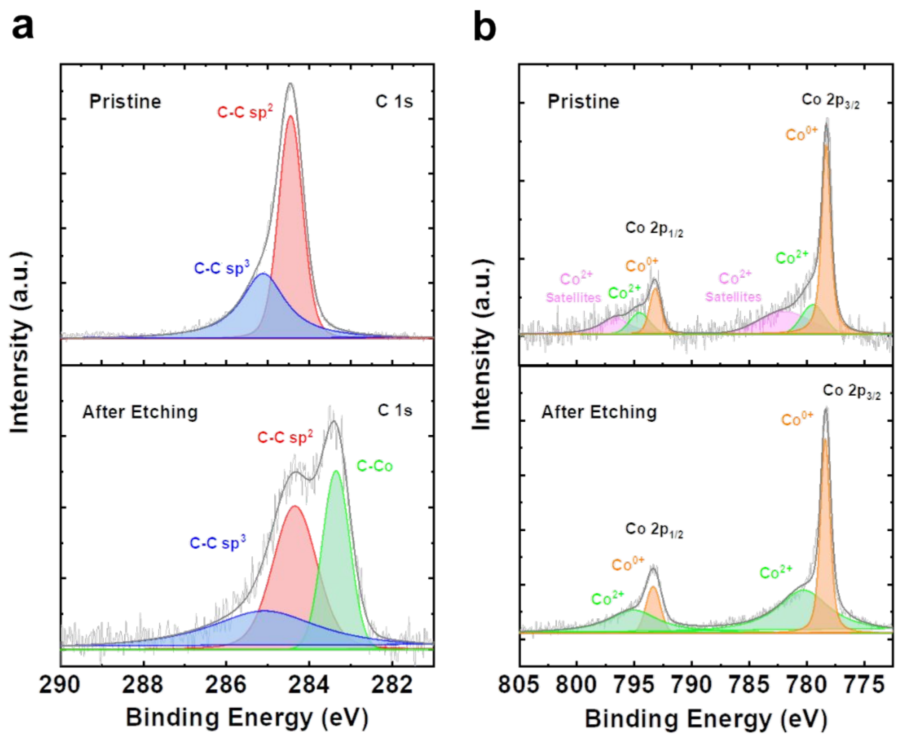

Figure S8. XPS spectrum of the GAA structure on the Co interconnect. (a) C 1s region (b) Co 2p region. The graphene was etched by the ion sputter to measure the Co and graphene interface.

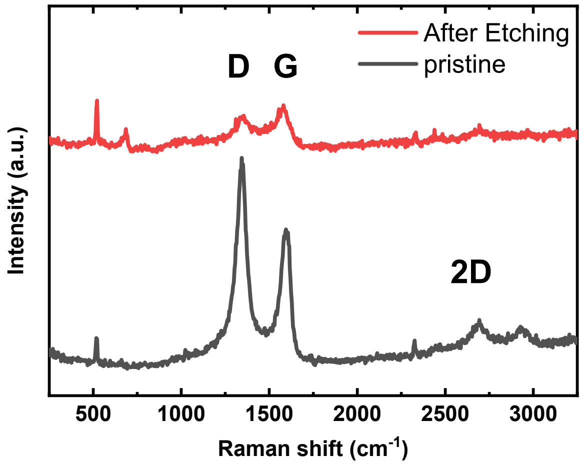

Figure S9. Raman Spectrum of before and after ion sputter for XPS measurement
